# The Ethics of Deep Brain Stimulation for the Treatment of Anorexia Nervosa

**DOI:** 10.1007/s12152-015-9240-9

**Published:** 2015-09-24

**Authors:** Hannah Maslen, Jonathan Pugh, Julian Savulescu

**Affiliations:** The Oxford Uehiro Centre for Practical Ethics, Suite 8, Littlegate House 16/17 St Ebbe’s Street, Oxford, OX1 1PT UK

**Keywords:** Deep brain stimulation, Anorexia Nervosa, Autonomy, Authenticity, Desires, Control, Emotions

## Abstract

There is preliminary evidence, from case reports and investigational studies, to suggest that Deep Brain Stimulation (DBS) could be used to treat some patients with Anorexia Nervosa (AN). Although this research is at an early stage, the invasive nature of the intervention and the vulnerability of the potential patients are such that anticipatory ethical analysis is warranted. In this paper, we first show how different treatment mechanisms raise different philosophical and ethical questions. We distinguish three potential mechanisms alluded to in the neuroscientific literature, relating to desire, control, and emotion, respectively. We explain why the precise nature of the mechanism has important implications for the patient’s autonomy and personal identity. In the second part of the paper, we consider practical dimensions of offering DBS to patients with AN in certain cases. We first discuss some limited circumstances where the mere offering of the intervention might be perceived as exerting a degree of coercive pressure that could serve to undermine the validity of the patient’s consent. Finally, we consider the implications of potential effects of DBS for the authenticity of the patient’s choice to continue using stimulation to ameliorate their condition.

There is some optimism amongst psychiatrists and neuroscientists that Deep Brain Stimulation (DBS) could be used effectively in the treatment of Anorexia Nervosa (AN). Indeed, there is emerging preliminary evidence from several case reports and investigational studies to support its effectiveness in some cases [1–4]. Although attention has been paid to the ethical issues raised by DBS in general [5], and to the use of non-invasive brain stimulation techniques for the treatment of eating disorders [6], there has not yet been a detailed analysis of the philosophical and ethical dimensions of using DBS to treat patients with AN. The analysis we provide here is important in a number of respects. Although familiar ethical concepts re-emerge in our discussion, the range of potential stimulation sites for DBS generates complexity, whereby some mechanisms have significant parallels with existing treatment options, whilst others permit no such comparison. Further, the differences between the various mechanisms require us to compare these distinct applications of the very same technology to evaluate which best promotes the patient’s interests.

AN is a life-threatening psychiatric disorder that is characterized by compulsive starvation and other weight loss behaviors, often resulting in severe emaciation, malnutrition and, in the most severe cases, death [7]. There is no medical consensus on the etiology of AN, and it is thought to emerge from a complex interaction between genetic, psychological and environmental factors, leading to both neurobiological and behavioral pathologies [8]. AN is notoriously difficult to treat, with current treatment options demonstrating only limited effectiveness. Against this backdrop of impenetrability, neuroscientists and psychiatrists have begun to explore the therapeutic prospects offered by techniques that directly intervene in the brain.

DBS is an invasive neurosurgical procedure that involves implanting electrodes into a target location of the patient’s brain. Electrical impulses are sent to the target location when the pulse generator (often implanted subcutaneously below the patient’s clavicle) is turned on. It is reversible both in the sense that the stimulation can be turned on or off, and in the sense that all the implanted components can be removed.

Although trials of DBS as a treatment for AN are still in early, investigational stages (and may not necessarily become an approved treatment), the invasive nature of the intervention and the vulnerability of the potential patients are such that anticipatory ethical analysis is warranted. In this paper, we shall first show how different treatment mechanisms raise different philosophical and ethical issues. Which of these potential mechanisms would promote an AN patient’s ability to direct their life and express their self in their decisions regarding their eating behavior, and which might potentially compromise these abilities, is in large part a philosophical question. We distinguish three potential mechanisms alluded to in the neuroscientific literature. Having distinguished these three mechanisms, we explain why they might have important implications for the patient’s autonomy and personal identity.

In the second part of the paper, we consider practical dimensions of offering DBS to patients with AN. Much of the debate regarding informed consent in this context has focused on whether severely anorexic patients can be competent to refuse treatment. Whilst these questions are undoubtedly important, they are not particular to consenting to the use of DBS, even though validly consenting to this treatment might arguably require that patients meet a higher standard of competence. We shall not address this question here. Rather, we shall discuss two potential hazards regarding obtaining valid informed consent specifically to the use of DBS in the treatment of AN, drawing on our earlier discussion of the mechanisms involved in the disorder. Although individual cases will vary depending on the severity of the illness and the characteristics and expressed wishes of the patients, we first discuss some limited circumstances where the mere offering of the intervention might be perceived as exerting a degree of coercive pressure that could serve to undermine the validity of the patient’s consent. Finally, we consider the implications of potential effects of DBS on a patient’s preferences for the authenticity of their choice to continue using stimulation to ameliorate their condition.

## Part One: Philosophical Issues Raised by the Mechanism of Action of DBS

In order to assess the moral challenges raised by the use of DBS in the treatment of AN, it is important to first explain how DBS differs from other interventions that have been used in the context of treating AN. According to Neil Levy’s ethical parity principle, internal and external interventions into the mind should be treated alike unless we can identify an ethically relevant difference between them [9]. In what ways does DBS differ from existing treatment methods that are employed in the context of AN? Following Focquaert and Schermer’s terminology [10], one obvious difference between DBS and some of the treatment methods used in the context of AN is that the former involves a *direct* manipulation of the subject’s brain, in that it changes the subject’s thought patterns and behavior *by virtue of* altering the subject’s brain structure and function.^1^ In contrast, other treatments used in the context of AN such as Cognitive Behavioural Therapy (CBT) and talk therapy are ‘indirect’, in so far as they first seek to change the subject’s thought patterns and behavior, and can only be said to alter the subject’s brain structure as result of effecting these changes.

Of course, the mere fact that DBS is direct and the other treatment methods mentioned above are indirect does not *itself* seem to be an ethically relevant difference [11]. However, we agree with Focquaert and Schermer that although the distinction between indirect and direct interventions is not itself morally relevant, it may nonetheless track a different distinction between, respectively, interventions that require *active* involvement from the subject, and interventions that subjects are *passive* with respect to [10]. They claim that this distinction *is* morally relevant, since direct brain interventions raise profound philosophical questions relating to personal identity and the autonomy of the recipients, because the recipients of such interventions are *passive* with respect to the changes that they undergo. More specifically, they claim that direct interventions are problematic with regards to the subject’s autonomy because the subject cannot deliberate and selectively endorse or reject the immediate and radical changes that such interventions bring about.

We broadly agree with this assessment, although we diverge from it in ways that we shall explain below. Furthermore, in the context of AN, we may observe that these issues become more complicated still, since we are considering the effects of such an intervention on an agent whose identity and apparent goals are to some extent bound up with their illness [12, 13].

Despite these potential ethical concerns, there is great interest in using DBS for patients with psychiatric disorders, including treatment-refractory AN, as it offers several advantages over existing treatment methods [8, 14]. Any intervention affecting feeling, experience, motivation and behaviour ultimately modifies activity in the brain. This is true of indirect psychological interventions, such as psychotherapy, as well as direct interventions, such as drugs, surgery or DBS; in some sense, they all involve the modification of brain states, so ethical issues raised by one potentially apply to others. That said, the ‘holy grail’ of neurointervention is precise control of the activation of single neurons producing targeted effects. All current interventions are crude ‘blunderbuss’ interventions affecting whole systems or large areas of the brain. DBS is the closest we currently get to precise targeting of neuronal activation in the brain. Indeed, probably the most controversial neurointervention would be to bring desire under close cognitive control. Choosing what to desire, and acting accordingly, would be an enormous power with implications for freedom, autonomy and well-being.

Although we are not yet at the stage where agents are enabled to simply will a desire into existence such that it motivates them sufficiently to act, DBS moves us much closer to this than we have ever been before. As we shall explain in the first part of this paper, in the context of AN, DBS could be used to impose a motivating desire to eat, or give patients the cognitive control required to resist compulsive motivation to engage in dangerous weight loss behavior. Such precise control is made possible because DBS can be used to selectively modulate the targeted brain regions proposed to be centrally involved in the pathophysiology. Moreover, stimulation can be adjusted according to individual patients’ demands, and stimulation is reversible. It is thus qualitatively and quantitatively different from most other neurointerventions and, in particular, deserves special treatment as ‘proof in principle’ of direct, brain based desire modification. Of course hypnotherapy, hormonal castration and many psychiatric interventions aim to bring desire under control but not under such, direct, nuanced control.

As we demonstrate below, DBS can have a wide range of different effects, and it is not clear that existing treatment methods can exert effects that are comparable to all of the ones that we describe. The effects that DBS will bring about depend in part upon the stimulation site and, correspondingly, the mechanism of action through which it operates. The scientific literature has suggested a range of such potential mechanisms. For example, a review conducted by McClelland et al. identifies a range of potential mechanisms:

Although this review reports solely on the effects of neuromodulation on eating-related behaviours and resulting weight gain/loss, such changes are likely to arise as a result of effects to some of the underlying cognitive, emotional and self-regulatory aspects of Eating Disorders, such as cognitive rigidity, impaired decision making, poor inhibition and altered self-control [15].

Similarly, Nestler outlines different ways in which improvements in the symptoms and prognosis of AN patients might be achieved:

The ability of DBS of these regions to induce some improvement in anorexia nervosa raises the question of whether such improvements occur primarily through the alleviation of obsessive-compulsive or depressive symptoms rather than the direct alleviation of abnormal feeding behavior. DBS of the subthalamic nucleus, a mainstay in the treatment of Parkinson’s disease, has been shown to cause weight gain in a subset of patients, raising the possibility that this region could be targeted for anorexia nervosa as well [16]

Understanding the mechanism of DBS will be of great significance in providing an adequate ethical analysis of the use of this technology in the context if AN. In this section, we identify three potential effects of DBS alluded to in the neuroscientific literature:

1. modification of aberrant reward processing,
2. increased control over (or reduction of) the drive towards compulsive behavior, and
3. regulation of aversive mood and affect.

The associated consequences for philosophical assessment are, correspondingly:

1. the imposition or amplification of a *desire* for food,
2. the promotion of comparative cognitive *control* over behavior, and
3. the modification of *emotional* symptoms or traits.

We shall begin by examining these three mechanisms, before going on to consider their implications for the patient’s autonomy and personal identity, as well as explaining how DBS raises ethical issues different to those raised by existing treatment methods in AN. Although we broadly agree with Focquaert and Schermer’s analysis of the morally relevant features of direct brain interventions, we shall nonetheless suggest that DBS could be used in the context of AN in a manner that serves to enhance the subject’s autonomy.

### The Imposition or Amplification of Desires: Liking and Wanting

Park and colleagues explain that a few, single-case reports on individuals with AN given DBS to the Nucleus Accumbens (part of the Ventral Striatum directly implicated in reward processing) suggest positive outcomes in terms of weight recovery and resolution of both anorexia and comorbid pathology [8]. Relatedly, the subthalamic nucleus has also been identified as a target for the modulation of reward processing [17]. Indeed, as Treasure and Schmidt report:

The importance of the subthalamic nucleus for the control of eating is shown by the emergence of binge eating as a possible side-effect of DBS in that region for the treatment of Parkinson’s disease [18].

The suggestion seems to be that stimulating a part of the brain implicated in attributing hedonic properties to food would increase the appeal of food and/or the motivation to eat.

In discussing this mechanism, it is important to be clear on some points of terminology. Park and colleagues point out that there is an important – and neurologically supported – distinction between ‘wanting’ and ‘liking’ in AN [8, 19]. Whilst ‘wanting’ is associated with motivational salience, ‘liking’ simply denotes hedonic pleasure taken in the object [8]. According to this distinction, if an agent *wants* something, they are to some degree motivated to pursue it, even though this motivation can be overridden by other competing motivations; however, they need not find it rewarding. In contrast, if an agent *likes* something, they find it rewarding but they need not be motivated to pursue it. Since rewardingness and motivation usually go hand in hand, both neurologically and phenomenologically, it does not usually create any conceptual confusion to presume that an agent who finds *x* rewarding is also motivated to pursue *x*. Indeed, in lay discussion, we often conflate ‘wanting’ and ‘liking’ under the umbrella term of ‘desiring’; if we say that an agent desires *x*, we often presume that they both like *x*, and that they are motivated to pursue *x*. However, for reasons elaborated below, we must be careful not to conflate the hedonic rewardingness involved in liking with the motivational salience involved in wanting under the umbrella term of ‘desiring’. This is especially so when discussing the aberrant neural functioning that can occur in AN. Where possible, we shall henceforth avoid using the term ‘desire’ in order to avoid this sort of conflation; however, we shall occasionally use the term ‘motivating desire’ to denote ‘wanting’.

It is also illuminating to draw an additional distinction between the explicit (conscious) and implicit (subconscious) levels at which wanting and liking can operate. Park and colleagues note that implicit ‘wanting’ can occur in the absence of cognitively driven, explicit wanting, or even in conflict with it [8]. For example, reaction time in a forced-choice procedure can provide an indirect measure of implicit wanting of food in the absence of explicit wanting, since our immediate reactions operate at a subconscious rather than conscious level [20].

The above terminological distinction is important because Park and colleagues argue that wanting and liking can cease to work in concert in AN [8]. When functioning properly, we tend to want (i.e., be motivated to pursue) things that we like (i.e., find pleasurable). In contrast, Park and colleagues present evidence to suggest that although the AN patient implicitly wants some types of food at a subconscious level (indirectly measured using reaction time in a forced-choice procedure), there is no explicit or implicit liking of food [8, 20]. Given the potential for such a disconnect, we should be careful to note that, according to this conceptualization of implicit wanting, there is a sense in which the AN patient can be motivated to pursue (at least at the implicit, subconscious level) something that she does not like. To explain how the anorexic patient manages this dissonance, Park and colleagues hypothesise that as self-starvation increases in AN, controlled processing amplifies to keep increased implicit ‘wanting’ of unsafe energy dense foods at bay as these threaten the pursuit of thinness which is central to AN [8, 21].

Therefore, the seemingly simplistic claim that DBS could be used to alter a desire for food is, in fact, a complicated proposition. Whilst increasing the rewardingness of food could bolster a patient’s motivation to eat, we should not assume that this will occur at the explicit, conscious level, nor that increasing implicit wanting (a subconscious motivating desire, on this framework) will guarantee that the wanted object will be liked, that is, experienced as pleasurable. The counterintuitive nature of this disjunction is an artifact of the aberrant reward processing in the brain of the anorexic patient. Given this picture, DBS mechanisms that might be described as simply acting on the patient’s ‘desires’ might in fact function in a number of different ways, and the precise function might have different implications for the patient’s autonomy, as we now explain.

#### Increasing Wanting

First, the intervention could make explicit an otherwise implicit want, thereby rendering it consciously accessible to the patient. In a sense, on this understanding, DBS might be understood to reveal to the patient what she actually already wants at the subconscious level. On many plausible accounts of autonomy, whether the patient identifies with or endorses this motivational desire (or want) at a higher-order level will be of great significance to whether the intervention works to promote their autonomy or frustrate it. On so-called ‘hierarchical’ accounts of autonomy, for an agent to be autonomous with respect to a motivating desire, they must endorse that desire with a second order volition that the first order desire in question be effective in moving them to act [22]. Alternatively, it might be claimed that autonomous agents must, *inter alia*, *rationally* endorse their first-order motivating desires. In turn, an agent can be said to rationally endorse a motivating desire if they maintain it on the basis of a belief that the object of that desire is *valuable*, or something that it would be good for them to attain [23–26]. For the purposes of brevity, we shall henceforth assume this latter, rationalist model. However, our arguments could readily be translated into the terms of hierarchical theories.

Sinnott-Armstrong and Pickard have drawn a similar distinction between wanting and liking in relation to addiction. They claim that ‘in the case of addiction, the theory is that addictive *wants* are triggered by drug-related cues that have become associated through sustained, heavy use with consumption’ [27]. However, despite this motivation, ‘some extreme addicts report no longer *liking* [gaining pleasure from] the drugs that they nonetheless *want* [are motivated to pursue]’. Indeed, Park and colleagues explicitly compare the AN patient’s behaviors and motivations with those involved in substance-dependence, where the mere cues associated with drug taking become associated with the hedonic properties of craving and taking drugs [8]. The rewardingness of drug taking essentially extends (or transfers) to drug related cues such that the cues are sufficient to motivate drug taking. There is a sense in which the drug addict is addicted to engaging in drug taking, not just the drugs themselves. In a similar way, Park et al. suggest that individuals with AN may ‘start to find that the reward of weight loss is no longer required as the now habitual weight loss behaviours themselves and associated cues have become rewarding or reinforcing’ [8]. The AN patient is ‘addicted’, so to speak, to engaging in weight loss behaviors, not just the weight loss itself. In the most extreme cases, weight loss behaviours will have become so reinforced that they cease even to be rewarding (liked) even when the AN patient feels compelled (wants) to persist with them.

However, there are interesting differences between drug addicts and anorexic patients in relation to the wanting/linking distinction, which illuminates an important consideration for using DBS to, alternatively, generate a ‘liking’ for food.

#### Increasing Liking

Consider the case of addiction. It seems that in many cases, the addict’s initial wanting to take a certain drug is often generated by the fact that they ‘like’ the hedonic effects of taking the drug. However, if the comparative hedonic effects of the drug decrease over time, the addict may get to the point at which they no longer ‘like’ the substance to which they are addicted, and come to form a motivational desire to quit. The problem for many addicts is that this desire to quit is often not sufficient to move them to action, often precisely because it comes into opposition with the more habitual motivation (i.e., the ‘want’) to seek out the substance to which they are addicted.

Contrast this to AN. It is not clear why sufferers of the disease initially start to become motivated to lose weight. However, it seems that some patients may in fact endorse their motivation to engage in severe weight loss, even in the absence of deriving pleasure from (liking) either the weight loss behaviors, or the weight loss to which they lead. This can occur when the anorexic patient values thinness as an end worth pursuing, an evaluation that admits of far more complexity than it being the case that they simply ‘like’ thinness. Once an addict no longer likes the substance to which they are addicted, there are rarely further non-hedonic *evaluative* grounds for their desire to continue taking the substance to which they are addicted. Their ‘want’ to nonetheless continue taking the drug in such a case may plausibly be understood as compulsive or habitual in a manner that is inimical to their exerting control over their substance-taking behavior [27]. In contrast though, the sufferer of AN often understands herself as having non-hedonic evaluative grounds for her desire to continue losing weight, even in the absence of deriving pleasure from these behaviours.

Relatedly, we can observe that, unlike in addiction, in AN there is often a goal beyond the compulsive or habitual behaviors themselves – i.e., that the weight loss behaviours constitute the carrying out of a long-term strategy, the aim of which is to become thinner. In contrast, in the case of addiction, although agents might use substances to experience pleasure, their substance use is not usually plausibly described as a strategy adopted for some independent long-term goal. So, whilst the AN sufferer pursuing thinness might have little cause for concern if their weight loss behavior became habitual in a manner that precluded their taking pleasure in individual instances of weight losing behavior (in so far as it will help them pursue the end that they value), it would not make sense for the addict to welcome the habituation of substance-use as instrumental for something they value. Indeed, such a difference corresponds to the observation made by Godier and Park that behavioural paradigms have ‘identified notable distinctions in information processing between AN and substance dependence [28] reflected in marked differences in the ability to delay reward in AN as compared to substance dependence’[29]. Addicts first and foremost pursue pleasure, whereas the person with AN often pursues a non-hedonic goal. This is why the cessation of liking (rewardingness) results in an addict’s frustration with their continued wanting (motivation) to use the substance, whilst this might not be true in the case of the person with AN.

These observations, distinguishing AN from cases of addiction, illuminate a potential concern with a DBS mechanism which imposes or amplifies the AN patient’s liking of food. Such a mechanism would make food more pleasurable, with the intention that the patient would become more motivated to pursue it; rewardingness and motivation would again work in concert. However, it is possible that an increased liking for food could come into conflict with the anorexic patient’s evaluative goals, even if the liking itself is not in direct conflict with another first-order motivation to engage in weight loss behaviors, or to achieve their effects. The problem is that the endorsement of being motivated to lose weight can persist amongst sufferers of AN even in the absence of their finding weight loss rewarding. Such patients regard the effects of extreme weight loss as something that they have reason to pursue, in so far as they believe that being thin is something that is good for them in a reason-implying sense; we may call such an assessment a positive rational evaluation.

We understand a rational evaluation to pertain to an agent’s apparent reasons, that is, reasons that agents understand themselves as having *given* their beliefs; accordingly, on this definition, an individual’s positive rational evaluation of x may not track objective facts about good-making features of x. This is explained by the fact that a rational evaluation of x will be partly grounded by P’s beliefs about x. To invoke Parfit’s terminology in this regard, we may say that P has a ‘merely apparent’ reason to want x, if the beliefs about x which partly ground her positive evaluation are false [30]. With this terminology in mind, we may say that a positive rational evaluation can reflect what an agent has only a ‘merely apparent’ reason to want. As such, on this understanding, P may form a positive rational evaluation for x, even if P actually has a strongly decisive reason to want to avoid x, given the actual facts about x.

We tend to treat positive rational evaluations as informing our practical deliberations; people want to live in accordance with what they believe they have reasons to do. That is not to say that an agent’s positive rational evaluations should always be respected; there may be good reasons not to respect some choices, even if they are grounded by a positive rational evaluation of the sort discussed.^2^ We lack the space to address this here; however, the important point regarding such evaluations for our purposes is that there can be a conflict between an individual’s positive rational evaluation of some goal, and what they are in fact motivated to do. A worry about using DBS in the context of AN is that it might generate such conflicts; DBS might cause patients to hold a first-order motivation to eat that is in conflict with a positive rational evaluation of excessive dieting. Such conflicts are likely to be highly distressing. Indeed, some of those involved in the research have warned that ‘Normalization of body weight after DBS for AN does not necessarily imply normalization of the distorted body image’ nor other psychological features associated anorexia [4]. They worry that DBS may have the consequence of increasing body weight without changing body image, and that ‘a “psychological hell” for the patient may result from this’ [4]. Whilst such an outcome has not yet been reported, the manipulation of first-order desires without attending to the patient’s evaluation of thinness presents a risk of psychological harm. Of course, it might be argued that existing indirect therapies could be used to mitigate the psychological harms associated with the changes effected by DBS. However, we shall raise some doubts about this below following our discussion of authenticity.

Having said this, and contrary to Focquaert and Schermer’s analysis of direct interventions more generally, it should be acknowledged that the imposition of a first-order liking for food could be beneficial for some AN patients' autonomy on some accounts, including the rationalist account that we briefly described above. For instance, suppose that a patient has reached an early stage of recovery in which they now believe that they have stronger reasons to ensure their continued survival than they have to pursue thinness; however, they still feel the pull of a first-order motivation to avoid food, and do not feel motivated to begin eating again. Qualitative interviews have revealed that such conflict is not an atypical experience, and can create a barrier to eating. Hope and colleagues describe how some patients, who had accepted treatment knowing that it would involve eating larger portions of food, were nonetheless unable to eat when the food was placed in front of them [31]. Hope and colleagues suggest that this inability is due in part to the conflict between a motivational desire to avoid significant health problems and the anxiety produced by the prospect of eating. In such cases, it might be claimed that amplifying a first-order motivational desire for food could serve to facilitate the individual’s autonomy by providing her with a first-order motivating desire that is in alignment with her rational evaluations.

### Promotion of Comparative Cognitive Control

In the previous section, we discussed a potential DBS mechanism that could amplify either an anorexic patent’s wanting or liking of food. This mechanism would seek to increase ‘healthy’ wants or likes that are deficient in the anorexic patient. However, an alternative hypothesized mechanism is that DBS may aid recovery by normalizing aberrant control over the compulsive wants that are characteristic of the disease. Outlining the neurocircuitry – specifically, a cortico-striatal thalamic circuit (CSTC) – involved, Park and colleagues explain that research suggests that different neural components are responsible for, on the one hand, the (bottom-up) *driving* of compulsive wanting behavior and, on the other hand, the (top-down) *control* or inhibition of this behavior [8]. Abnormalities in either of these components (hypoactivity/hyperactivity) may result in an increase in compulsive behaviours.

Drawing comparisons with the behavior exhibited in obsessive-compulsive disorder (OCD), Park and colleagues hypothesise that anorexic patients might suffer from hyperactive weight loss compulsion. Although weight loss behaviors might start out as goal-driven, these behaviours themselves (as opposed to their effects on weight) become rewarding and are thereby reinforced until they become habitual motivations. Consequently, habitual weight loss behavior becomes dissociated from the original goals and is difficult to exert control over. Indeed, this hypothesis is borne out in the experiences of AN patients. Hope and colleagues use qualitative data to demonstrate how the sense of lack of control can ‘creep up on’ the AN patient [31]. They describe how a patient might engage in pathological weight loss behavior unaware of the dangers, but by the time they become concerned, they ‘find the behavior has taken hold’.

Thus, in both the neuroscientific and qualitative discussions, the argument seems to be that what starts out as goal-driven becomes habit driven, in a way that is difficult to resist. Extending the comparison with OCD, Park and colleagues note that ‘Symptomatic alleviation in treatment resistant OCD and addictions following DBS targeted within the CSTC circuit […] supports the involvement of these circuits in compulsivity. There are preliminary suggestions that this circuit may indicate potential targets for DBS for severe enduring AN’ [8].

We should note that there are therefore two ways in which comparative cognitive control could be increased: either the compulsive want to diet and avoid eating could itself be reduced, or the top-down control over this compulsion could be increased. Consider first the reduction of the compulsive want. Where the weight loss behaviors themselves have taken on the rewarding properties originally associated with successful weight loss itself, the patient’s inability to cease engaging in weight loss is principally a result of the strength of the motivating desire to continue to engage in these behaviours; the want is too strong to resist. An intervention that reduced or inhibited this bottom-up motivation would essentially ‘down grade’ a compulsive want to a mere want, which could then be overridden by other competing motivations (e.g., to survive). Now consider the increase of top-down control. This mechanism suggests that the aberrant function – the principal factor leading to compulsive behavior – is a failure of top-down control. Indeed, Fineberg et al. suggest that failures in cortical control of fronto-striatal neural circuits may underpin compulsivity in a number of psychiatric disorders [32] and Meunier et al. suggest that patients with related conditions (OCD and substance dependence) show significant impairments in suppressing ongoing behavior and corresponding significant reduction of right orbitofrontal connectivity [33]. An intervention that improved top-down control would not affect the strong want to engage in weight loss per se, but the increase in ability to resist may forestall acting compulsively, such that the want is no longer effectively compulsive, despite persisting.

To understand the implications of these two mechanisms for the patient’s autonomy, reconsider first the reduction of the compulsive want. It seems that such an intervention could promote the patient’s autonomy, if their weight loss is driven by irresistible reinforced habits that no longer align with the patient’s autonomously chosen goals. Recall the example at the end of the previous section. We suggested that one way of facilitating such a patient’s autonomy would be to impose a motivating desire for food. Suppose now that the patient in question already had a motivating desire for food that they rationally endorsed, but that it was being overridden by a much stronger compulsive motivating desire to avoid eating. In such a scenario, DBS could be used to reduce the strength of a compulsive motivating desire towards the latter over-learnt behaviours, behaviours that no longer serve the patient’s goals. The consequence would be that – having been reduced in absolute strength – the patient’s motivating desire to engage in weight loss can be overridden by her motivating desire to survive.

On the other hand, if a patient had formed a positive rational evaluation of the motivation to engage in weight loss behaviors, they might not welcome a reduction of their wanting to do what serves their valued end. On the contrary, they are likely to object to an intervention that would reduce the power of a motivational drive that is highly conducive to their being able to achieve their goal.

In contrast, it seems that the alternative possibility – whereby top-down control is increased without affecting the absolute strength of the compulsive motivational drive – would only serve to promote the agent’s autonomy, as it would be up to the agent whether or not to exert this control. Although the motivational desire itself is left intact, remedying a failure in top-down cortical control would give the patient the resources to be more successfully self-governing, even in the face of strong drives. The patient regains control over her actions, and can decide whether or not to resist the motivational desire. This would be akin to the person who is able to resist drinking nonetheless choosing to drink in some, even many, situations. Again, in the context of AN, it seems that the use of a direct brain intervention could in some cases be used to enhance the patient’s autonomy.

### Modification of Emotional Symptoms or Traits

The final mechanism we consider is drawn from a study that examined the effects of using DBS to modify emotional traits of patients with anorexia. Lipsman et al. believe that anorexia is ‘predominantly a disorder of emotional processing’ on the grounds that it is, according to their model, primarily the limbic structures that are implicated in the disorder. Anorexia, they point out is a disorder ‘marked by high rates of depressed mood and affective dysregulation’ [34].

Lipsman and colleagues stimulated the subcallosal cingulate, which has a key role in modulating emotional states and projects cortically, to medial- and orbitofrontal cortex, as well as subcortically to nucleus accumbens. They reported as follows:

Our initial study in a small group of treatment-refractory patients (**N**.6; average age: 38 years; average illness duration: 18 years) showed DBS to be reasonably safe, and associated with improvements in comorbid mood and anxiety symptoms [34].

Although they found that three of their six patients had achieved and maintained a BMI greater than their historical baselines after 9 months, Lipsman and colleagues speculated that these effects were achieved indirectly via a primary effect on mood, anxiety and affective regulation. Improved mood, they suggest, enhanced the uptake and effectiveness of conventional anorexia treatment, which consequently lead to increases in weight [34].

The plausibility of such a mechanism also corresponds with patient experiences. Interviews conducted by Hope and colleagues demonstrate the doubly impairing effects of anxiety on the AN patient’s rational assessment and on their agency vis-à-vis eating [31]. With respect to beliefs and decision-making, Hope et al. show how aversive affective responses to body size lead the patient to discount the objective evidence of low weight, such that beliefs grounded on this evidence are not given the appropriate epistemic weight. The high levels of anxiety generated by seeing her body, and not the direct perception of it, ‘tell’ the AN patient that she is fat.

Anxiety also impairs agency; it makes it very difficult for the patient to eat in the same way that arthritis makes it difficult for an arthritic man to walk, even though there is a sense in which he can. Hope and colleagues therefore conclude that

[E]ating to put on weight is also very difficult because of the extreme anxiety and feelings of self-disgust that accompany the intake of food, particularly intake that is perceived as excessive. This anxiety is not only unpleasant in itself, but also gives the message that eating, and weight gain, are dangerous [31].

An intervention that reduces anxiety and other aversive affect leaves the patient’s drives and desires less directly affected, although there is plausibly a relationship between how we feel and what we are motivated to do. In cases where the intervention acts on an agent’s mood and emotions, the intervention may raise concerns pertaining to the patient’s narrative identity, that is the qualitative sense of identity that we invoke when we discuss the continuity of a person’s character over time [35].

Whilst it seems intuitively plausible to claim that raised mood and decreased anxiety are the sorts of changes that people will welcome, mood and emotions can be closely bound up with our sense of qualitative identity in ways that clinicians should at least be aware of. The following quotation from a study conducted by Tan et al. illustrates this in relation to AN [12]:

*Interviewer*: If your anorexia nervosa magically disappeared, what would be different from right now?

*Participant*: Everything. My personality would be different.

*Interviewer*: Really!

*Participant*: It’s been, I know it’s been such a big part of me, and—I don’t think you can ever get rid of it, or the feelings, you always have a bit—in you. […]

*Interviewer*: Let’s say you’ve got to this point, and someone said they could wave a magic wand and there wouldn’t be anorexia any more.

*Participant*: I couldn’t.

*Interviewer*: You couldn’t.

*Participant*: It’s just a part of me now.

*Interviewer*: Right. So it feels like you’d be losing a part of you.

*Participant*: Because it was my identity. (Participant I).

Thus, the AN patient’s anxiety and associated false beliefs about body image can become central to their sense of identity. Whilst this does not provide an all things considered argument against a treatment that modifies affect, the possibility that the patient may experience herself as undergoing significant change should at least be brought to her attention in the process of obtaining consent.

We pointed out above that Wu et al. warn against the ‘psychological hell’ that DBS might expose anorexic patients to, and suggested that it might be argued that existing indirect therapies could be used to mitigate these psychological harms. However, considerations of authenticity give us reason to believe that indirect therapies may offer only limited mitigation of the harms discussed here; if DBS brings about a change in the patient’s qualitative identity, even if indirect therapies may help the patient come to terms with this change after it occurs, they will not serve to bring back the patient’s sense of identity that was once so important to them. Instead, such therapies rely on the active involvement with who the patient is now, in a qualitative sense. Furthermore, even if these harms could plausibly be mitigated, we surely have some moral reason to employ DBS in a way that avoids such psychological harms if possible, rather than seek to mitigate them once they have occurred.

The use of DBS to reduce anxiety prompts relevant comparisons with using antidepressants or anti-anxiety medications. To a large extent, the ethical issues associated with this particular mechanism – especially those associated with authenticity – are not unique to DBS, since these pharmacological agents also amount to direct interventions. For example, philosophers have debated whether taking the drug Prozac to treat depression would promote or diminish the authenticity of the agent’s experiences [36].

In contrast, there is little precedent in the context of AN with which to draw comparisons concerning the direct amplification a desire for food, nor the direct increase of comparative control over compulsive weight loss behaviors. Thus, DBS raises unique issues associated with the potential mechanisms and effects in these cases and, perhaps more saliently, raises the broader issue of how we should choose between these mechanisms. Whilst relative effectiveness, in the sense of promoting weight gain, will be a significant consideration when opting to implement one mechanism instead of another, the likely effects on the patient’s sense of identity and their ability to be self-governing should also factor into the clinician-patient decision.

For example, as we discussed above, a mechanism that increases the patient’s capacity for top-down cognitive control over compulsive behaviours would be a paradigm example of an intervention that increases the recipient’s capacity for autonomous decision-making, even though it is brought about via a direct intervention. Similarly, a mechanism that modulates affect in the way that DBS is hypothesised to do may sometimes promote autonomy; by reducing the anxiety which sometimes prevents patients from assigning the correct epistemic weight to facts about their low weight, and from engaging in healthier eating behaviours [31] . To the extent that this is the case, the *voluntary* decision to undergo DBS could also constitute a paradigm instance of someone living authentically, in line with the existentialist view, according to which living authentically is to consciously shape one’s own characteristics.

Although there are clearly ways in which DBS is able to increase the patient’s capacity for autonomous decision-making, this will not *necessarily* be the case. For example, it may be detrimental to alter bottom-up motivating desires that were not endorsed by the patient at a higher-order level. Thus, even where the specifics of some individual mechanisms invoke familiar ethical debates, the decision about which site is all things considered the best to target requires new comparisons, which encompass the physical effects, as well as the mechanism-specific effects on the patient’s ability to be self-governing.

## Part Two: Obtaining Valid Consent and Assessing Patient Competence

### Perceived Coercion and Validity of Consent to Undergo DBS

Much of the ethical debate surrounding the treatment of AN has focused on the question of whether anorexic patients are competent to refuse treatment, and whether their right to refuse treatment should be respected [12, 13, 23, 37–44]. We lack the space to settle these questions here. Instead, we shall assume that AN patients can be competent to refuse treatment, and suggest that giving a competent, severely anorexic patient the option of treatment in the form of DBS could still present ethical problems regarding consent if appropriate safeguards are not put in place. We shall consider two problems; the first relates to the potential for coerced choice, the second relates to the authenticity of the patient’s choice to continue using DBS after it may already have affected her evaluative judgments.

#### Perceived Coercion

In limited cases, severely anorexic patients are subjected to involuntary force-feeding; although this is an invasive and potentially traumatic procedure, it is sometimes necessary to prevent the patient’s death. Subjecting an AN patient to involuntary force-feeding amounts to compulsion in Feinberg’s sense of the term, where compulsion is understood to amount to exerting direct force to make a particular option impossible [45]. Although compulsory force-feeding is sometimes permitted, there are good reasons for supposing that compulsory DBS would not be similarly permitted; it is more invasive, riskier and poses a greater threat to the mental life of the patient.

Compelling a competent patient to undergo involuntary force-feeding violates their autonomy in an obvious way. Moreover, even if the patient was incompetent, it could be a distressing and terrifying event associated with significant harm (though arguably necessary to save life).

Accordingly, it seems that the most plausible way in which DBS treatment might be incorporated into the care of AN patients is as a voluntary alternative to other treatments. However, a potential problem with this is that a patient’s belief in the possibility of being subjected to involuntary treatments such as force-feeding may have more subtle effects on the validity of their consent to undergo other treatments. To see why, we may begin by contrasting the compulsion involved in force-feeding with Feinberg’s understanding of coercion; for Feinberg, coercion does not make an option impossible (as is the case in compulsion), but rather destroys its appeal by increasing its cost [45]. A paradigm case is that of the highway-man who tells their victim that he will shoot them unless they hand over their wallet. The victim here is coerced; although it is possible for him not to hand over his wallet, this option lacks the appeal it once had by virtue of the fact that there are now large costs associated with taking that option. Crucially for our purposes here, coercion is commonly understood to invalidate consent [46].

With this in mind, we might notice that if a patient believes (rightly or wrongly) that there is a credible threat of being subjected to involuntary force-feeding if they do not agree to some other therapeutic intervention, then they may consent to the latter intervention only because they strongly want to avoid the former. This, however, bears the hallmarks of coercion. Indeed, Tan’s studies have suggested that AN patients often perceive themselves as being coerced in this way as a part of their treatment. Consider the following reports from another of Tan’s studies [47], which addressed anorexic patients’ attitudes towards forced treatment:

I was meant to go as an inpatient at the P [adolescent general psychiatric unit] about 2 years ago, but I didn’t want to, and so I did the treatment at home. And I didn’t really think I had a choice [about whether to have treatment], looking back on it now I think: “Well, why didn’t I just not eat?” And I didn’t want to, but I still did it because I thought I didn’t have a choice. So in a way that was sort of forced upon me, I didn’t want to get better then. 19P

I mean I came, technically I came in here voluntarily, and technically I suppose it was my decision …but it doesn’t always feel like that when there’s a lot of pressure and a lot of guilt, that’s played a big part in it. And personally I was just left feeling that …there really was no other choice. 39P

Arguably then, it may be the case that coercive pressure is sometimes placed on competent patients’ choices in this sort of manner [13, 48, 49]. Having said this, and although we lack the space to further develop the thought here, *prima facie*, it seems that invalidating a patient’s consent by placing such coercive pressure on a patient’s choice is morally preferable to subjecting her to involuntary treatment; the use of compulsion, it seems, may plausibly involve further moral wrongs that the use of coercion does not.

We have suggested that even if AN patients are sometimes compelled to undergo involuntary force-feeding, there are good reasons not to compel patients to undergo involuntary DBS treatment. Accordingly, it seems that the most plausible way in which DBS treatment might be incorporated into the care of AN patients is as a voluntary alternative to other treatments. However, the above discussion should make it clear that this may not be sufficient to dispell all of the potential concerns regarding patient autonomy and informed consent in this context. The problem is that if the patient justifiably or even falsely believes that a failure to give consent to DBS will result in doctors resorting to involuntary force-feeding further down the line, they might opt for the neurosurgical intervention in order to avoid the trauma of involuntary force-feeding. However, whilst we may be prepared to allow the coercion of AN patients to get them to enter into existing treatment programmes, it is not clear that our moral reasons here will be powerful enough to justify coercing patients to undergo a riskier and deeply invasive procedure such as DBS.

In response to this observation, it might be claimed that patients would not feel coerced to undergo DBS in this way because they would also be given the option of choosing to eat before being offered the options of choosing to undergo DBS or being force-fed. Offering DBS, it might be claimed, should be a last resort before compelling force-feeding.

Yet, it is not clear that this approach would be sufficient to dispel the concern that we are raising. The problem with it is that the anorexic patient may believe that undergoing DBS is preferable to actually eating food, because they may be under the impression that they will be able to continue resisting any new or enhanced urges to eat. Consider the following, again from Tan’s study:

*Interviewer*: What do you think about being admitted to hospital for treatment against your will?

*Participant*: If I didn’t want to, if I was really really in my losing weight frame of mind, it’s the last thing I would want to be doing; and I don’t think it would be very successful because I’d be fighting against it.

A patient’s ability to ‘fight’ against their treatment depends a great deal on the particular treatment in question. Whilst a patient may correctly believe that she can fight against her doctors by refusing to engage with indirect treatments that require her active involvement, it is less clear that a patient is correct to believe that she can be successful in fighting against forced feeding. The key question for our purposes here is what the anorexic patient is likely to believe about their ability to ‘fight’ against the changes that a direct intervention such as DBS would precipitate – if they believe that they are able to ‘fight’ mentally against it in a manner that they are unable to fight physically against force-feeding, they may think that DBS treatment is preferable to consuming food, whether voluntarily or involuntarily. The reason for this is that both of these latter options directly frustrate the anorexic patient’s central desire to refrain from eating. Whilst the patient may be correct to believe that DBS would not have this direct effect, she may not adequately attend to the power of the urges that DBS might bring about, or to the other morally relevant aspects of the treatment that must be sufficiently understood if her consent to the treatment is to be truly valid. At very least, a full explanation of the potential manner in which DBS might act in AN is necessary for the patient’s consent to be valid.

As such, it seems that there are some plausible grounds for the concern that patients may perceive that coercive pressure is being placed on their choice, whether or not such pressure is being intentionally applied. This is arguably problematic with regard to the quality of their consent to undergoing the procedure, since their decision is made in the perceived context of a ‘forced choice’, or coercion, and this may be understood to invalidate consent. Whilst we do not claim that it is impossible to autonomously consent to DBS for AN, we raise this concern as one that should be considered in cases where patients might be likely to perceive the situation as less than fully free. Patients might ideally want to forgo both food and DBS, and not be force-fed. But that option might not be available; and whilst non-consensual force-feeding may already be permitted in some limited cases, there are good reasons for refraining from using DBS in the absence of *valid* consent.

Having said that, there may be ways in which we might mitigate these concerns. First, we might only allow the use of DBS following an independent treatment review; part of the review panel’s role could be to clarify for the patient that they are not being coerced into accepting treatment. One way in which the patient might be assured of this is to ensure that they understand that the option of force-feeding will not be taken. If medical professionals undertook not to force-feed, even to save life, then the perception that this would be done if DBS was not accepted would be removed. Thus, paradoxically, DBS might be most ethical in contexts where force-feeding is not a possibility.

### Reversibility, Authenticity and Shared Decision-Making

One notable feature of DBS is that it would be possible to give patients control over their stimulation – whether to continue it and how to fine-tune it – in consultation with their physician. On the face of it, this is empowering; patients control their treatment. However, we should not simply assume that this enhances authenticity or respects autonomy. Stimulation, as we discussed, could be used to impose a change that is inauthentic. The mere fact that an agent finds something rewarding and pursues it on this basis, does not guarantee that she values or rationally endorses it. Reward can generate strong motivation, and the fact that a person continues to stimulate a part of the brain implicated in reward does not *necessarily* mean that the choice is an expression of reflection, deep-seated values, or that agent’s authentic self.

To illustrate the problem in the context of AN, suppose that an anorexic patient validly consents to undergoing DBS. Whilst she is experiencing the effects of the stimulation, suppose that she embraces the change in desires that the stimulation precipitates, and tells her care team that she endorses the changes, and would like to undergo further stimulation in the future. However, when stimulation is stopped and the effects wear off, she is horrified by the changes that occurred whilst she was experiencing the effects of stimulation, and withdraws her consent to continue treatment.^3^

This possibility adds a further layer of uncertainty to existing difficulties in determining the authentic wishes of the AN patient. Hope and colleagues provide qualitative evidence to show that AN patients often experience a ‘shifting between mind sets’ [31]: in one mind set, the patient has a set of beliefs, goals and affective states that she sees as directing her to eat more and gain weight. In the other mindset, the patient panics and feels she is not able to eat to put on weight. In this mindset, she may not even be sure that to be healthy she needs to gain weight. Thus, there is an instability and ambivalence that leads us to question whether one of the two ‘mindsets’ can be identified as the one that should be respected, as the ‘authentic’ self, or perhaps neither, or even elements of both. The possibility of adding a further ‘self’ (the ‘stimulated self’) to compete for authenticity renders determination of which preference to respect even more difficult.

However, although we do not have the space to develop a detailed position here, we would in fact oppose practical employment of the concept of authentic selves in such contexts – with the associated assumption that one self is the ‘true’ self – recommending instead that physicians consider the pattern and stability of an agent’s preferences over time, both on and off stimulation. Such a diachronic approach avoids the automatic prioritization of preferences under treatment.

Indeed, it might be tempting to suppose that we should respect the wish to continue treatment that the patient expressed when she was under the influence of DBS. In support of this claim, it might be argued that the desires that underlie the patient’s choice to now refuse treatment (i.e., in the absence of the effects of DBS) have a ‘pathological origin’, and do not reflect what the agent authentically desires. For instance, Tan and colleagues have suggested that the paramount importance that anorexic patients place on thinness can be understood to be pathological in so far as this sort of evaluation is closely identified with the diagnostic criteria of AN [12].

However, it seems that this claim requires further support. As Craigie notes, Tan et al.’s proposal seems in some way to beg the question; after all, the inclusion of this sort of evaluation in the diagnostic criteria of AN *itself* seems to presuppose that the evaluation is pathological [23]. In the absence of further justification of why the anorexic patient’s desire to refuse treatment is inauthentic, physicians should not make the substantive claim that only a patient’s desire to continue treatment is authentic. Whilst a patient might express this sort of desire under the influence of DBS, we should be wary that this technology can have profound effects on patient’s drives, emotions and values. Yet it is possible that the changes that the stimulation brings about may alienate the agent from what one authentic self rationally endorses. This raises deep questions about the nature of competence to consent and authenticity; does the patient reveal what she authentically wants when she is under the influence of DBS, when she is not under the influence of DBS, or neither? Must valid consent be based on an authentic desire?

We cannot adequately address these questions here, and can again only gesture towards an account that does not identify distinct selves, but looks at the patient’s preferences and values over time. It is imperative that physicians encourage patients to reflect on the changes both when ‘on’ and ‘off’ stimulation to better determine whether the patient embraces them as authentic over time. If we believe that the use of DBS is only permissible with the valid consent of the patient, we must answer these deeper questions about consent and authenticity, and not merely make the substantive presumption that the patient must always ‘really’ want to get better, and that we should only take into account the patient’s expression of *this* desire.

## Conclusion

DBS is undoubtedly a powerful new medical technology, and existing studies suggests that it has at least some promise as a potential new therapy for AN. Further, as we have demonstrated, DBS raises a novel ethical issue in this context in so far as we must choose between implementing various mechanisms of the very same technology, a choice that requires reflection not just on their relative effectiveness at promoting weight gain, but also on their implications for the patient’s ability to govern herself.

Of course, a comprehensive ethical analysis of the use of this technology should focus first on the quality of the patient’s consent to undergo the intervention. In this paper, we have assumed, for the sake of discussion, that the AN patient could be competent with respect to their decision to accept (or refuse) treatment, and that these decisions should be respected. It is worth noting that this is a somewhat controversial assumption in this context; however, by making this assumption, we have been able to explore further ethical considerations that are specific to the use of DBS to treat AN. It also requires us to compare potential target sites along a number of ethically relevant dimensions.

We have suggested that whilst the way in which DBS manipulates various neural processes might plausibly enhance the patient’s ability to make autonomous decisions with regards to her eating behavior, the use of this technology to alter first-order motivating desires or the patient’s emotional traits may confer significant harms as well as potential benefits. The various ways in which different mechanisms are likely to affect AN patients’ experiences of themselves, and their ability to be self-governing must be borne in mind in research and clinical development protocols for DBS treatment. Furthermore, practitioners should be wary of the new ways in which the use (and indeed, mere prospect) of DBS treatment could introduce new avenues of perceived coercion, and new problems regarding the authenticity of AN patients’ desires regarding their continued treatment and eating behaviors.

## Footnotes

1 We do not claim that DBS is the only direct treatment method used in the context of DBS. For instance, pharmacological agents may also be direct in this way.

2 For instance, if an agent’s positive rational evaluation is based on a manifestly false belief that is held in an epistemically irrational manner, that might plausibly give us grounds for not respecting a choice based on that evaluation. Indeed, although rationalist theories of autonomy claim that a necessary condition of an agent’s being autonomous with respect to a motivating desire is that they rationally endorse the object of that desire, such theories might also stipulate a further necessary condition of autonomy pertaining to the epistemic rationality of the beliefs undergirding the agent’s decision. This a nuanced debate that we cannot explore here; however, it should be noted that we invoke the concept of a positive rational evaluation here to highlight the possibility of psychological harms rather than to make a point about autonomous decision-making or what sort of choices should be respected in the context of AN.

3 This scenario has been raised elsewhere [5].
